# Supplementary material for: Failure of the Brain Glucagon-Like Peptide-1-Mediated Control of Intestinal Redox Homeostasis in a Rat Model of Sporadic Alzheimer’s Disease
Source: Antioxidants (Basel). 2021 Jul 13;10(7):1118. doi: 10.3390/antiox10071118 (PMC8301063; doi:10.3390/antiox10071118)
Supplement: Supplementary file 1 [file antioxidants-10-01118-s001.zip › Supplement 1.pdf]

Homolak et al. (2021) Failure of the brain GLP-1-mediated control of intestinal redox homeostasis in a rat model of sporadic Alzheimer's disease

Supplement 1

Table S1. Plasma SOD – main effects model

contrast	estimate	SE	df	lower.CL	upper.CL	t.ratio	p.value
CTR - STZ	2.5185	0.5706	36	1.3613	3.6757	4.4140	0.0001
(Ex-9) - SAL	-0.6483	0.5706	36	-1.8054	0.5089	-1.1362	0.2634

Table S2. Plasma SOD – treatment interaction model

contrast	estimate	SE	df	lower.CL	upper.CL	t.ratio	p.value
(CTR Ex-9) - (STZ Ex-9)	1.3197	0.7800	35	-0.7838	3.4232	1.6920	0.3430
(CTR Ex-9) - CTR SAL	-1.7840	0.7592	35	-3.8314	0.2634	-2.3500	0.1061
(CTR Ex-9) - STZ SAL	1.8703	0.7592	35	-0.1771	3.9176	2.4636	0.0837
(STZ Ex-9) - CTR SAL	-3.1037	0.7800	35	-5.2071	-1.0002	-3.9793	0.0018
(STZ Ex-9) - STZ SAL	0.5506	0.7800	35	-1.5529	2.6540	0.7059	0.8941
CTR SAL - STZ SAL	3.6542	0.7592	35	1.6069	5.7016	4.8136	0.0002

Table S3. plasma TBARS – main effects model

contrast	estimate	SE	df	lower.CL	upper.CL	t.ratio	p.value

CTR - STZ	-0.0082	0.0051	36	-0.0186	0.0022	-1.6004	0.1183
(Ex-9) - SAL	-0.0140	0.0051	36	-0.0244	-0.0035	-2.7201	0.0100

Table S4. Plasma TBARS – interaction model

contrast	estimate	SE	df	lower.CL	upper.CL	t.ratio	p.value
(CTR Ex-9) - (STZ Ex-9)	-0.0083	0.0075	35	-0.0284	0.0118	-1.1143	0.6833
(CTR Ex-9) - CTR SAL	-0.0140	0.0073	35	-0.0336	0.0055	-1.9353	0.2322
(CTR Ex-9) - STZ SAL	-0.0222	0.0073	35	-0.0417	-0.0026	-3.0528	0.0214
(STZ Ex-9) - CTR SAL	-0.0057	0.0075	35	-0.0258	0.0144	-0.7694	0.8677
(STZ Ex-9) - STZ SAL	-0.0138	0.0075	35	-0.0340	0.0063	-1.8571	0.2648
CTR SAL - STZ SAL	-0.0081	0.0073	35	-0.0277	0.0115	-1.1175	0.6813

Table S5. Plasma NRP – main effects model

contrast	estimate	SE	df	lower.CL	upper.CL	t.ratio	p.value
CTR - STZ	2906783	877107.3	36	1127926	4685639	3.3141	0.0021
(Ex-9) - SAL	-3295336	877107.3	36	-5074192	-1516480	-3.7571	0.0006

Table S6. Plasma NRP – interaction model

contrast	estimate	SE	df	lower.CL	upper.CL	t.ratio	p.value
(CTR Ex-9) - (STZ Ex-9)	2296262.9	1267209	35	-1121275.9	5713801.6	1.8121	0.2849
(CTR Ex-9) - CTR SAL	-3873723.3	1233410	35	-7200111.2	-547335.4	-3.1407	0.0172
(CTR Ex-9) - STZ SAL	-388553.6	1233410	35	-3714941.5	2937834.3	-0.3150	0.9890
(STZ Ex-9) - CTR SAL	-6169986.2	1267209	35	-9587524.9	-2752447.4	-4.8690	0.0001
(STZ Ex-9) - STZ SAL	-2684816.5	1267209	35	-6102355.2	732722.3	-2.1187	0.1671
CTR SAL - STZ SAL	3485169.7	1233410	35	158781.8	6811557.6	2.8256	0.0371

Homolak et al. (2021) Failure of the brain GLP-1-mediated control of intestinal redox homeostasis in a rat model of sporadic Alzheimer's disease

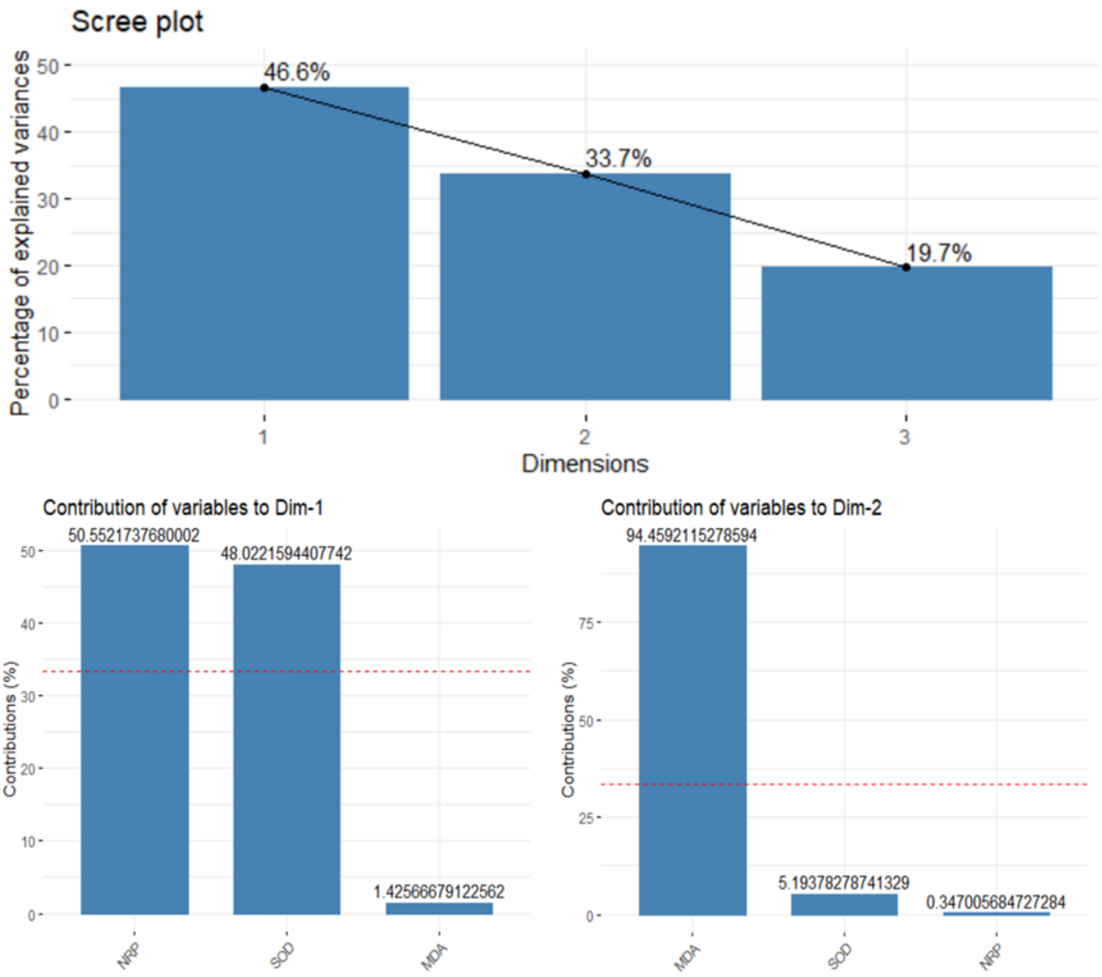

Figure S1. Scree plot and contribution of individual variables to dimensions for plasma oxidative stress PCA.

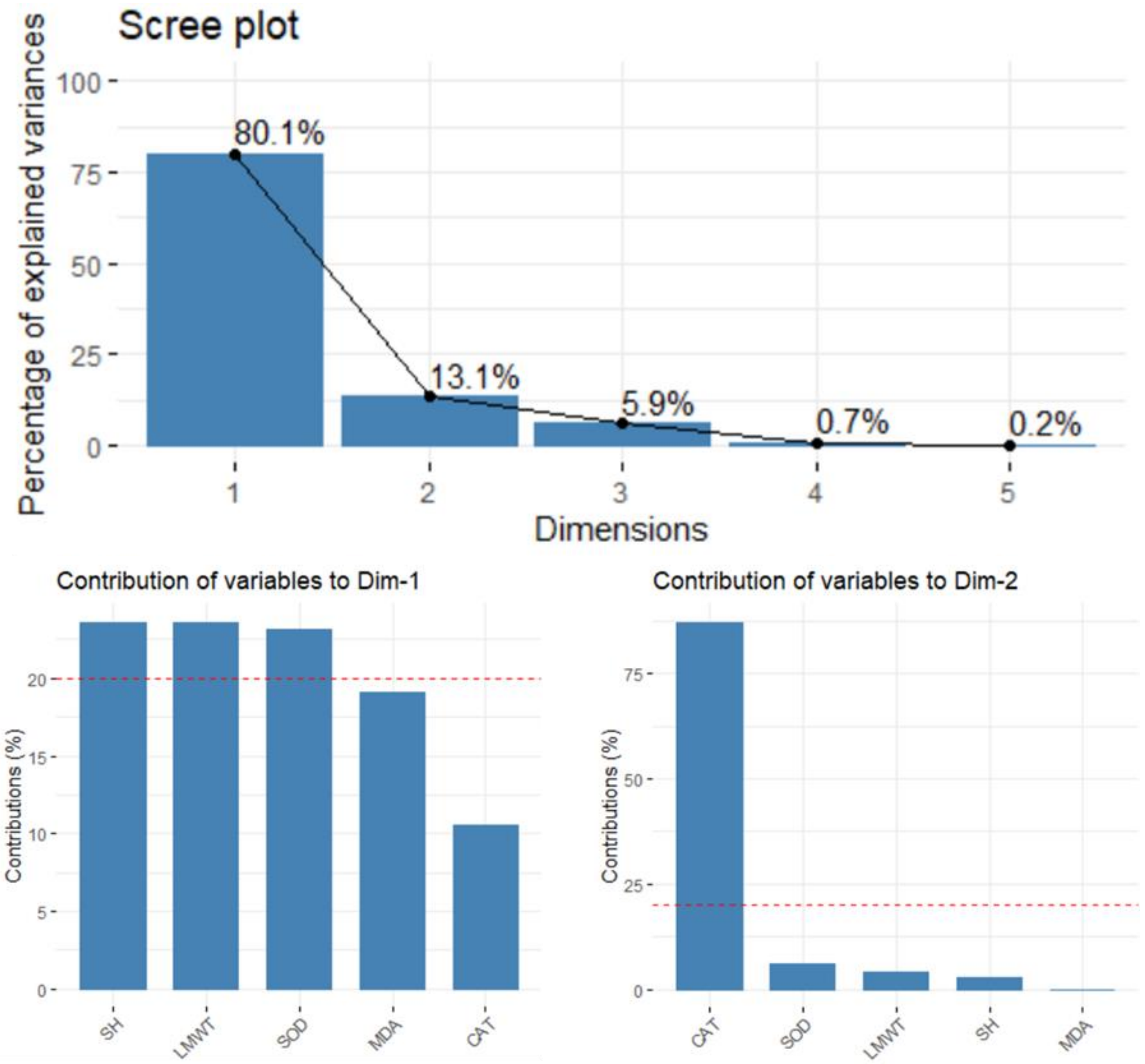

Figure S2. Scree plot and contribution of individual variables to dimensions for duodenum oxidative stress PCA.

Table S7. Duodenum oxidative stress PCA position relative to Dim 1 – main effects model

contrast	estimate	SE	df	lower.CL	upper.CL	t.ratio	p.value
CTR - STZ	-0.7609	0.931	16	-2.7345	1.2128	-0.8172	0.4258
(Ex-9) - SAL	0.9501	0.931	16	-1.0236	2.9238	1.0205	0.3227

Table S8. Duodenum oxidative stress PCA position relative to Dim 1 – interaction model

Homolak et al. (2021) Failure of the brain GLP-1-mediated control of intestinal redox homeostasis in a rat model of sporadic Alzheimer's disease

contrast	estimate	SE	df	lower.CL	upper.CL	t.ratio	p.value
(CTR Ex-9) - (STZ Ex-9)	1.1843	1.2199	15	-1.4159	3.7846	0.9708	0.3470
(CTR Ex-9) - CTR SAL	2.6792	1.1502	15	0.2276	5.1307	2.3293	0.0342
(CTR Ex-9) - STZ SAL	0.1892	1.1502	15	-2.2623	2.6408	0.1645	0.8715
(STZ Ex-9) - CTR SAL	1.4948	1.2199	15	-1.1054	4.0951	1.2253	0.2394
(STZ Ex-9) - STZ SAL	-0.9951	1.2199	15	-3.5954	1.6051	-0.8157	0.4274
CTR SAL - STZ SAL	-2.4899	1.1502	15	-4.9415	-0.0384	-2.1648	0.0469

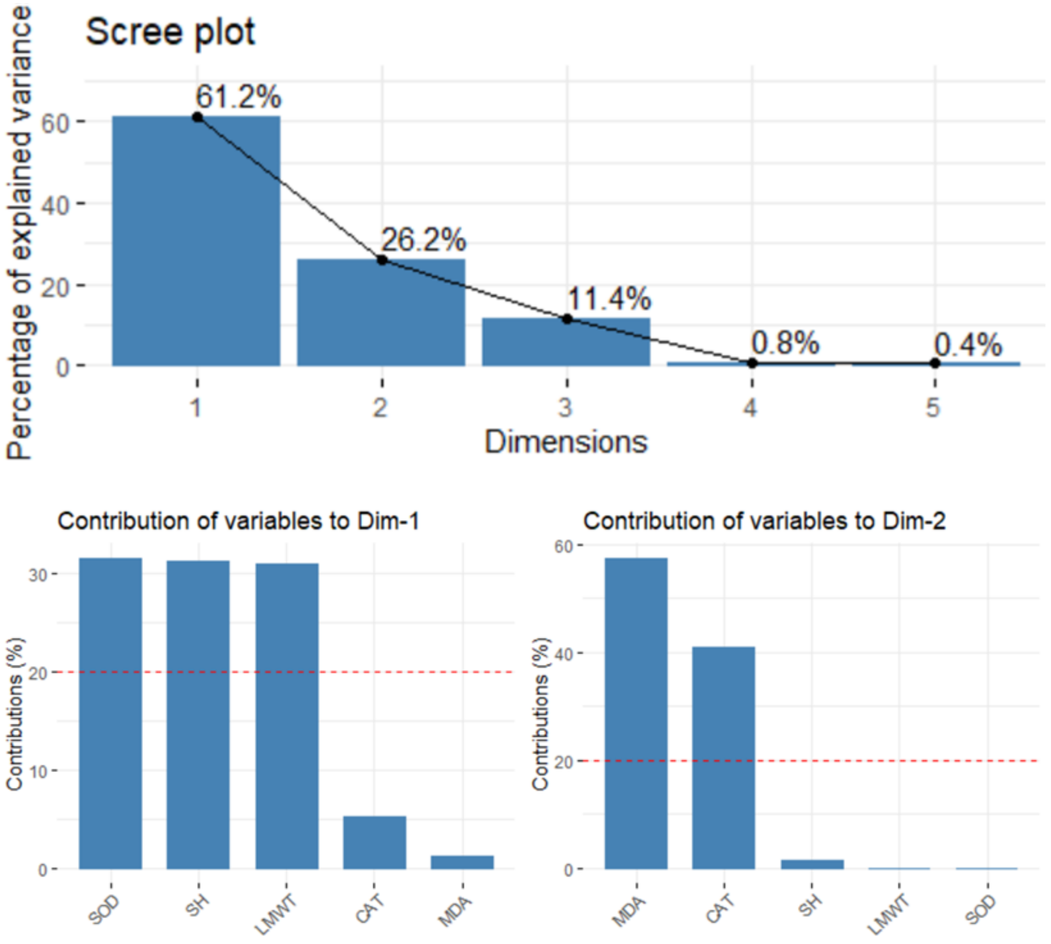

Figure S2. Scree plot and contribution of individual variables to dimensions for ileum oxidative stress PCA.

Table S9. Ileum oxidative stress PCA position relative to Dim 1 – main effects model

contrast	estimate	SE	df	lower.CL	upper.CL	t.ratio	p.value
CTR - STZ	0.6091	0.7543	20	-0.9643	2.1825	0.8075	0.4289
(Ex-9) - SAL	-0.0920	0.7543	20	-1.6654	1.4814	-0.1220	0.9041

Table S10. Ileum oxidative stress PCA position relative to Dim 1 – interaction model

contrast	estimate	SE	df	lower.CL	upper.CL	t.ratio	p.value

(CTR Ex-9) - (STZ Ex-9)	0.8147	1.0671	19	-1.4187	3.0482	0.7635	0.4545
(CTR Ex-9) - CTR SAL	0.1342	1.1192	19	-2.2082	2.4766	0.1199	0.9058
(CTR Ex-9) - STZ SAL	0.5171	1.0671	19	-1.7163	2.7505	0.4846	0.6335
(STZ Ex-9) - CTR SAL	-0.6805	1.1192	19	-3.0230	1.6619	-0.6081	0.5503
(STZ Ex-9) - STZ SAL	-0.2976	1.0671	19	-2.5311	1.9358	-0.2789	0.7833
CTR SAL - STZ SAL	0.3829	1.1192	19	-1.9596	2.7253	0.3421	0.7360

Table S11. Duodenum TBARS – main effects model

contrast	estimate	SE	df	lower.CL	upper.CL	t.ratio	p.value
CTR - STZ	2.1665	16.8437	16	-33.5407	37.8736	0.1286	0.8993
(Ex-9) - SAL	5.9182	16.8437	16	-29.7889	41.6254	0.3514	0.7299

Table S12. Duodenum TBARS – interaction model

contrast	estimate	SE	df	lower.CL	upper.CL	t.ratio	p.value
(CTR Ex-9) - (STZ Ex-9)	50.8383	18.5679	15	11.2617	90.4149	2.7380	0.0153
(CTR Ex-9) - CTR SAL	49.1821	17.5060	15	11.8689	86.4953	2.8094	0.0132
(CTR Ex-9) - STZ SAL	8.0847	17.5060	15	-29.2285	45.3979	0.4618	0.6508
(STZ Ex-9) - CTR SAL	-1.6562	18.5679	15	-41.2328	37.9204	-0.0892	0.9301
(STZ Ex-9) - STZ SAL	-42.7536	18.5679	15	-82.3302	-3.1770	-2.3026	0.0360
CTR SAL - STZ SAL	-41.0974	17.5060	15	-78.4106	-3.7842	-2.3476	0.0330

Table S13. Duodenum LMWT – main effects model

contrast	estimate	SE	df	lower.CL	upper.CL	t.ratio	p.value
CTR - STZ	3.5732	2.8087	16	-2.3810	9.5274	1.2722	0.2215
(Ex-9) - SAL	-4.0849	2.8087	16	-10.0391	1.8693	-1.4544	0.1652

Table S14. Duodenum LMWT – interaction model

contrast	estimate	SE	df	lower.CL	upper.CL	t.ratio	p.value
(CTR Ex-9) - (STZ Ex-9)	-1.6827	3.7951	15	-9.7717	6.4064	-0.4434	0.6638

(CTR Ex-9) - CTR SAL	-8.7568	3.5780	15	-16.3832	-1.1304	-2.4474	0.0272
(CTR Ex-9) - STZ SAL	-0.5117	3.5780	15	-8.1381	7.1148	-0.1430	0.8882
(STZ Ex-9) - CTR SAL	-7.0741	3.7951	15	-15.1632	1.0149	-1.8640	0.0820
(STZ Ex-9) - STZ SAL	1.1710	3.7951	15	-6.9180	9.2601	0.3086	0.7619
CTR SAL - STZ SAL	8.2451	3.5780	15	0.6187	15.8715	2.3044	0.0359

Table S15. Duodenum SH – main effects model

contrast	estimate	SE	df	lower.CL	upper.CL	t.ratio	p.value
CTR - STZ	2.3588	2.1714	16	-2.2445	6.9620	1.0863	0.2935
(Ex-9) - SAL	-2.9370	2.1714	16	-7.5402	1.6663	-1.3526	0.1950

Table S16. Duodenum SH – interaction model

contrast	estimate	SE	df	lower.CL	upper.CL	t.ratio	p.value
(CTR Ex-9) - (STZ Ex-9)	-1.2986	3.0005	15	-7.6940	5.0969	-0.4328	0.6713
(CTR Ex-9) - CTR SAL	-6.1879	2.8289	15	-12.2176	-0.1582	-2.1874	0.0450

(CTR Ex-9) - STZ SAL	-0.5782	2.8289	15	-6.6079	5.4515	-0.2044	0.8408
(STZ Ex-9) - CTR SAL	-4.8893	3.0005	15	-11.2848	1.5061	-1.6295	0.1240
(STZ Ex-9) - STZ SAL	0.7204	3.0005	15	-5.6751	7.1158	0.2401	0.8135
CTR SAL - STZ SAL	5.6097	2.8289	15	-0.4200	11.6394	1.9830	0.0660

Table S17. Duodenum CAT – main effects model

contrast	ratio	SE	df	lower.CL	upper.CL	t.ratio	p.value
CTR / STZ	0.9714	0.5584	16	0.2872	3.2856	-0.0504	0.9604
(Ex-9) / SAL	0.7011	0.4030	16	0.2073	2.3713	-0.6178	0.5454

Table S18. Duodenum CAT – interaction model

contrast	ratio	SE	df	lower.CL	upper.CL	t.ratio	p.value
(CTR Ex-9) / (STZ Ex-9)	3.8914	2.7691	15	0.8538	17.7346	1.9094	0.0755
(CTR Ex-9) / CTR SAL	2.4072	1.6150	15	0.5760	10.0590	1.3093	0.2101
(CTR Ex-9) / STZ SAL	0.6811	0.4570	15	0.1630	2.8461	-0.5724	0.5755

(STZ Ex-9) / CTR SAL	0.6186	0.4402	15	0.1357	2.8192	-0.6750	0.5100
(STZ Ex-9) / STZ SAL	0.1750	0.1246	15	0.0384	0.7977	-2.4491	0.0271
CTR SAL / STZ SAL	0.2829	0.1898	15	0.0677	1.1824	-1.8818	0.0794

Table S19. Duodenum SOD – main effects model

contrast	estimate	SE	df	lower.CL	upper.CL	t.ratio	p.value
CTR - STZ	0.0207	0.0236	16	-0.0294	0.0708	0.8745	0.3948
(Ex-9) - SAL	-0.0379	0.0236	16	-0.0880	0.0122	-1.6018	0.1288

Table S20. Duodenum SOD – interaction model

contrast	estimate	SE	df	lower.CL	upper.CL	t.ratio	p.value
(CTR Ex-9) - (STZ Ex-9)	-0.0158	0.0331	15	-0.0864	0.0549	-0.4760	0.6409
(CTR Ex-9) - CTR SAL	-0.0703	0.0313	15	-0.1369	-0.0036	-2.2480	0.0400
(CTR Ex-9) - STZ SAL	-0.0172	0.0313	15	-0.0838	0.0494	-0.5500	0.5904
(STZ Ex-9) - CTR SAL	-0.0545	0.0331	15	-0.1251	0.0162	-1.6434	0.1211

(STZ Ex-9) - STZ SAL	-0.0014	0.0331	15	-0.0721	0.0692	-0.0425	0.9667
CTR SAL - STZ SAL	0.0531	0.0313	15	-0.0135	0.1197	1.6980	0.1101

Table S21. Ileum TBARS – main effects model

contrast	estimate	SE	df	lower.CL	upper.CL	t.ratio	p.value
CTR - STZ	8.1165	9.1806	20	-11.0339	27.2670	0.8841	0.3872
(Ex-9) - SAL	20.1114	9.1806	20	0.9610	39.2619	2.1906	0.0405

Table S22. Ileum TBARS – interaction model

contrast	estimate	SE	df	lower.CL	upper.CL	t.ratio	p.value
(CTR Ex-9) - (STZ Ex-9)	26.5062	11.4890	19	2.4595	50.5529	2.3071	0.0325
(CTR Ex-9) - CTR SAL	40.3400	12.0497	19	15.1197	65.5604	3.3478	0.0034
(CTR Ex-9) - STZ SAL	28.2279	11.4890	19	4.1812	52.2746	2.4570	0.0238
(STZ Ex-9) - CTR SAL	13.8339	12.0497	19	-11.3865	39.0543	1.1481	0.2652

(STZ Ex-9) - STZ SAL	1.7217	11.4890	19	-22.3250	25.7685	0.1499	0.8825
CTR SAL - STZ SAL	-12.1121	12.0497	19	-37.3325	13.1083	-1.0052	0.3274

Table S23. Ileum CAT – main effects model

contrast	ratio	SE	df	lower.CL	upper.CL	t.ratio	p.value
CTR / STZ	1.3681	0.3664	20	0.7826	2.3918	1.1704	0.2556
(Ex-9) / SAL	1.8305	0.4902	20	1.0470	3.2001	2.2575	0.0353

Table S24. Ileum CAT – interaction model

contrast	ratio	SE	df	lower.CL	upper.CL	t.ratio	p.value
(CTR Ex-9) / (STZ Ex-9)	1.2282	0.4642	19	0.5568	2.7090	0.5439	0.5928
(CTR Ex-9) / CTR SAL	1.6256	0.6444	19	0.7091	3.7267	1.2258	0.2352
(CTR Ex-9) / STZ SAL	2.5043	0.9465	19	1.1354	5.5236	2.4290	0.0252
(STZ Ex-9) / CTR SAL	1.3236	0.5246	19	0.5774	3.0342	0.7072	0.4880
(STZ Ex-9) / STZ SAL	2.0390	0.7706	19	0.9244	4.4973	1.8852	0.0748
CTR SAL / STZ SAL	1.5405	0.6106	19	0.6720	3.5316	1.0902	0.2893
